# Microtubule polyglutamylation and acetylation drive microtubule dynamics critical for platelet formation

**DOI:** 10.1186/s12915-018-0584-6

**Published:** 2018-10-18

**Authors:** Juliette van Dijk, Guillaume Bompard, Julien Cau, Shinji Kunishima, Gabriel Rabeharivelo, Julio Mateos-Langerak, Chantal Cazevieille, Patricia Cavelier, Brigitte Boizet-Bonhoure, Claude Delsert, Nathalie Morin

**Affiliations:** 1 Universités de Montpellier, 34293 Montpellier, France; 2 CRBM, CNRS, UMR 5237, 1919 Route de Mende, 34293 Montpellier, France; 3 IGH, CNRS UMR9002, 141, rue de la Cardonille, 34396 Montpellier, France; 4 Montpellier Rio Imaging, 34293 Montpellier, France; 5 Department of Advanced Diagnosis, National Hospital Organization Nagoya Medical Center, 4-1-1 Sannomaru, Naka-ku, Nagoya, 4600001 Japan; 6 Present address: Department of Medical Technology, Gifu University of Medical Science, Seki, Gifu 5013892 Japan; 7 INM, INSERM UMR1051, 34293 Montpellier, France; 8 IGMM, CNRS, UMR 5535, 1919 Route de Mende, 34293 Montpellier, France; 9 3AS Station Expérimentale d’Aquaculture Ifremer, Chemin de Maguelone, 34250 Palavas-les-Flots, France

**Keywords:** Microtubules, Acetylation, Polyglutamylation, Tubulin isotype, Platelets, Megakaryocytes, αIIbβ3 integrin, CHO cells

## Abstract

**Background:**

Upon maturation in the bone marrow, polyploid megakaryocytes elongate very long and thin cytoplasmic branches called proplatelets. Proplatelets enter the sinusoids blood vessels in which platelets are ultimately released. Microtubule dynamics, bundling, sliding, and coiling, drive these dramatic morphological changes whose regulation remains poorly understood. Microtubule properties are defined by tubulin isotype composition and post-translational modification patterns. It remains unknown whether microtubule post-translational modifications occur in proplatelets and if so, whether they contribute to platelet formation.

**Results:**

Here, we show that in proplatelets from mouse megakaryocytes, microtubules are both acetylated and polyglutamylated. To bypass the difficulties of working with differentiating megakaryocytes, we used a cell model that allowed us to test the functions of these modifications. First, we show that α2bβ3integrin signaling in D723H cells is sufficient to induce β1tubulin expression and recapitulate the specific microtubule behaviors observed during proplatelet elongation and platelet release. Using this model, we found that microtubule acetylation and polyglutamylation occur with different spatio-temporal patterns. We demonstrate that microtubule acetylation, polyglutamylation, and β1tubulin expression are mandatory for proplatelet-like elongation, swelling formation, and cytoplast severing. We discuss the functional importance of polyglutamylation of β1tubulin-containing microtubules for their efficient bundling and coiling during platelet formation.

**Conclusions:**

We characterized and validated a powerful cell model to address microtubule behavior in mature megakaryocytes, which allowed us to demonstrate the functional importance of microtubule acetylation and polyglutamylation for platelet release. Furthermore, we bring evidence of a link between the expression of a specific tubulin isotype, the occurrence of microtubule post-translational modifications, and the acquisition of specific microtubule behaviors. Thus, our findings could widen the current view of the regulation of microtubule behavior in cells such as osteoclasts, spermatozoa, and neurons, which express distinct tubulin isotypes and display specific microtubule activities during differentiation.

**Electronic supplementary material:**

The online version of this article (10.1186/s12915-018-0584-6) contains supplementary material, which is available to authorized users.

## Background

Megakaryocytes (MKs) are highly differentiated cells whose function is to assemble and release platelets in the blood stream. The production of platelets from mature megakaryocytes occurs via the extension of long thin and branched cytoplasmic processes named proplatelets [1–3]. Microtubules (MTs), one of the main structural components of proplatelets, are absolutely required for the extension of proplatelets [4]. The driving force for proplatelet extension is not driven by MT polymerization but results from the bundling of MTs and their dynein-dependent sliding past each other [5]. At the distal end of the proplatelets, the MT bundle coils and re-enters the shaft forming swellings with a unique circular MT ring [6]. These swellings are severed and released into the blood stream as platelets or bigger circular platelet intermediates called preplatelets [1]. An elegant study showed that preplatelets can reversibly change to barbell-shaped platelet structures by twisting their MT ring. Barbell structure, containing a platelet-sized MT coil at each end, is further cleaved in two platelets [1]. The MT ring persists in the periphery of the platelets and is known as the marginal band. It exerts forces that maintain the discoid shape of the resting platelet [7]. MT transitions observed during platelet activation could result from molecular motors that drive marginal band elongation and induce its supercoiling/buckling [8]. However, visco-elasticity of the marginal band itself allows it to adapt its shape. Indeed, a recent modeling study shows that a rapid increase in cortical tension, too fast for MT cross linker reorganization, overcomes MT bundle rigidity and induces marginal band supercoiling [9].

Given the crucial role of the MT network, one key step is to understand the spatio-temporal regulation of MT dynamics in these giant cells. MT-binding proteins regulate MT functions. Interactions of these MT-binding proteins with MTs are controled by both tubulin isotype content and post-translational modifications (PTMs) of MTs [10, 11]. An hematopoietic-specific tubulin isotype, tubulin beta1 class VI (β1tubulin), is expressed only in late mature megakaryocytes and accounts for 90% of the total beta tubulin pool in platelets [12]. *TUBB1*−/− mice suffer from thrombocytopenia and extensive bleeding [13]. Their platelets have defective marginal bands with reduced MT coils pointing to specific functions of β1tubulin in platelet structure and functions. In humans, TUBB1 mutations are also linked to macrothrombocytopenia, altered MT dynamics and/or assembly [14–17]. Mechanisms that confer specific functions to this tubulin in MT coiling are not yet understood. Whether MT PTMs are present in megakaryocytes is not yet known, but the marginal band of resting platelets is heavily acetylated, and platelet activation induces a massive MT deacetylation, which is mediated by the tubulin deacetylase HDAC6 [18].

Beautiful live imaging strikingly revealed MT dynamics in proplatelets and platelet intermediates (reviewed in [19]). Nevertheless, the role of PTMs in the regulation of MT behavior has not been addressed due to the difficulties inherent to differentiate megakaryocytes in vitro and to the absence of a reliable cell model.

Platelet membrane complex, GPIb-IX-V, and platelet fibrinogen receptor, integrin αIIbβ3, regulate proplatelet formation and platelet release [20, 21], although the molecular mechanisms involved remain poorly understood.

Several years ago, a Chinese hamster ovary (CHO)-based cell model was developed [22]. The expression of partially activated αIIbβ3D723H integrins in these cells induced fibrinogen-dependent, MT-driven proplatelet-like extensions (PPLLs) [22, 23]. Yet, the capacity of these cytoplasmic elongations to induce MT rearrangements similar to megakaryocyte-derived proplatelets was not investigated. Here, we characterized this cell model in depth. We show that αIIbβ3D723H integrin signaling nicely recapitulates PPLL elongation, swelling formation, and severing accompanied with MT bundling, coiling, and marginal band-like formation. We used this model to highlight the importance of MT acetylation and polyglutamylation during proplatelet-like elongation.

## Results

### β1tubulin-containing MTs of proplatelets extending from megakaryocytes are acetylated and polyglutamylated

**Fig. 1:**
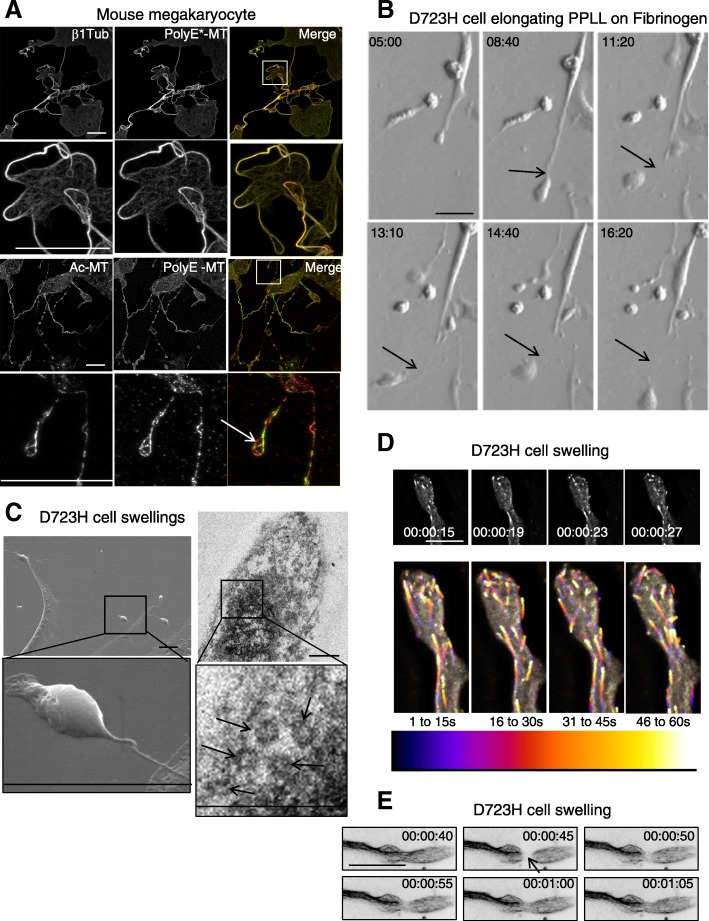
Proplatelet MTs are acetylated and polyglutamylated. D723H cells on fibrinogen induce PPLLs containing dynamic MTs. **a** Maximum intensity projections (MIP) of mature mouse megakaryocytes extending proplatelets stained with β1tubulin or modified MT antibodies. Ac-MT and PolyE-MT patterns are not identical. Low and high magnifications (insets) are shown. Bars 25 μm (**b**). Modulation contrast time course in hours of cytoplast cleavage at the end of a PPLL extension occurring in D723H cells spread on fibrinogen. Arrows point to the thinning and severing site of the extending protrusion. Bar 30 μm. **c** Left panels, scanning electron microscopy micrograph of PPLL extending from a D723H cell spread on fibrinogen, showing swellings at their end (top) and detail of a swelling arising from an extremely thin cytoplasmic bridge (bottom). Bars 5 μm. Right panels, transmission electron microscopy micrograph of dense material in a D723H cell swelling (top), higher magnification shows that 5–7 MT bundles are present in the swelling. Bars 500 nm. **d**, **e** Fast wide field imaging. **d** 3D stacks (five slices) derived from live GFP-EB3 comets imaged every second at the tip of a PPLL (MIP). Bar 10 μm. Color temporal projection of MIP indicates comets evolution during temporal windows (1–15, 16–30, 31–45, and 46–60s) and shows MTs enter the forming swellings and coiling back in the shaft of the PPLL. **e** Live 2D widefield imaging of Sir tubulin-labeled MTs. MT dynamics are observed after FRAPping (for 5 s) the MTs in the PPLL shaft. Bar 20 μm

Microtubule PTMs in megakaryocytes are not well characterized. We found that β1tubulin isotype containing microtubules (MTs) are highly polyglutamylated (PolyE-) and acetylated (Ac-) in extending proplatelets of mouse embryonic liver-derived megakaryocytes (Fig. 1a). In some instances, discrete differences of PolyE- and Ac-tubulin staining could be observed (Fig. 1a, arrow).

In order to address whether these MT PTMs have specific functions during proplatelet elongation, we took advantage of a previously described Chinese hamster ovary cell line (D723H cells). These cells express a constitutive but partially activated mutant αIIbβ3 integrin (β3D723H mutation) which upon binding to its fibrinogen ligand, induces MT-dependent elongation of one or two cytoplasmic branches (PPLL) that form swellings at their growing ends [22, 24]. However, whether D723H cells can recapitulate MT behavior observed in megakaryocyte-derived proplatelets or allow the formation of platelet-like cytoplasts was not reported.

### Partial activation of αIIbβ3 integrin signaling in CHO cells recapitulates MT bundling, MT coiling, and cytoplast severing observed in differentiating MKs extending proplatelets

As previously described [22], when spread on fibrinogen, D723H cells formed PPLL extensions with dynamic swellings at their tips while αIIbβ3 wild-type-expressing cells (WT cells) were devoid of these protrusions (Additional file 1: Figure S1A, Additional file 2: Movie S1, Additional file 3: Movie S2). Using live microscopy, we observed the occurrence of swelling cleavage and release of cytoplasts in the cell culture medium (Fig. 1b, Additional file 4: Movie S3). To better visualize these structures, we used electron microscopy. PPLL extensions are flat and become thinner toward their growing ends that can harbor one or several bulbous swellings (Fig. 1c, left panels). Unlike initially reported [22], we found that these swellings are colonized by bundled MTs (Additional file 1: Figure S1B). Typically, several MT coils were observed in thin-section electron micrographs of the swellings (Fig. 1c, right panels, arrows) in agreement with the 7–12 MT coils reported for platelets [25].

GFP-EB3/mCherry-lifeAct live cell imaging showed that swelling’s shape evolves with time. mCherry-lifeAct reveals the dynamic cortical actin ring while GFP-EB3 marks growing MT plus tips (Additional file 1: Figure S1C). Fluorescent tubulin live imaging confirms our electron microscopy data and shows that MTs in the swellings are bundled (Additional file 1: Figure S1D, Additional file 5: Movie S4). Some of the MTs in the bundles are highly dynamic as seen by imaging EB3 comets every second. EB3 comets enter the swellings and turn around while some reenter the branch shaft, as better visualized using colored time projections (Fig. 1d, Additional file 6: Movie S5). To further confirm that MT bundles that enter a newly forming swelling are dynamic, we used fluorescent recovery after photobleaching (FRAP) (Fig. 1e, Additional file 7: Movie S6) which shows an example of fluorescent tubulin reincorporated as soon as 5 s after MT bleaching. Finally, we observed that D723H cells produce barbell-like structures containing dynamic EB3 comets (Additional file 1: Figure S1E, Additional file 8: Movie S7), similar to well-described barbell platelet intermediates formed during megakaryocyte differentiation and platelet release.

Our data show that αIIbβ3 integrin engagement in D723H cells is sufficient to induce a behavior that closely resembles proplatelet elongation upon megakaryocyte differentiation and that is characterized by the dynamic behavior of PPLL MTs together with MT bundling/coiling occurring in the swellings and ultimately the severing of these swellings.


Additional file 2: Movie S1. WT cell spreading on fibrinogen. Modulation contrast time-lapse microscopy images were acquired every 6 min. (AVI 2124 kb)



Additional file 3: Movie S2. D723H cells spreading on fibrinogen. Modulation contrast time-lapse microscopy images were acquired every 6 min. (AVI 2512 kb)



Additional file 4: Movie S3. D723H cell spreading on fibrinogen, cytoplast cleavage. Modulation contrast time-lapse microscopy images were acquired every 10 min. (AVI 2356 kb)



Additional file 5: Movie S4. Swelling of a D723H cell spreading on fibrinogen contains coiled MTs. Cells were incubated with Sir-Tubulin overnight before spreading for 16 h and imaged every 2 s for 2 min using a high speed wide field fluorescence microscope. (AVI 896 kb)



Additional file 6: Movie S5 Swelling of a D723H cell spreading on fibrinogen with dynamic GFP-EB3 comets. Cells were transfected with GFP-EB3 plasmid overnight before spreading for 16 h and imaged every sec for 1 min (5 planes/time point) using a high speed wide field fluorescence microscope. (AVI 1358 kb)



Additional file 7: Movie S6. MT in a swelling of a D723H cell spreading on fibrinogen rapidly recover after FRAP. Cells were treated as in movie S4 and imaged every 5 s for 2 min (5 planes/time point) using a high speed wide field fluorescence microscope. (AVI 1146 kb)



Additional file 8: Movie S7. A D723H cell-derived barbell-like platelet contain dynamic MTs. Cells were treated as in movie S5 and imaged every sec for 1 min (5 planes/time point) using a high speed wide field fluorescence microscope. (AVI 1250 kb)


### Acetylation and polyglutamylation decorate MT bundles and the marginal band-like structure during PPLL elongation

We further used this unique cell model to study the importance of MT PTMs during PPLL elongation and platelet release.

**Fig. 2:**
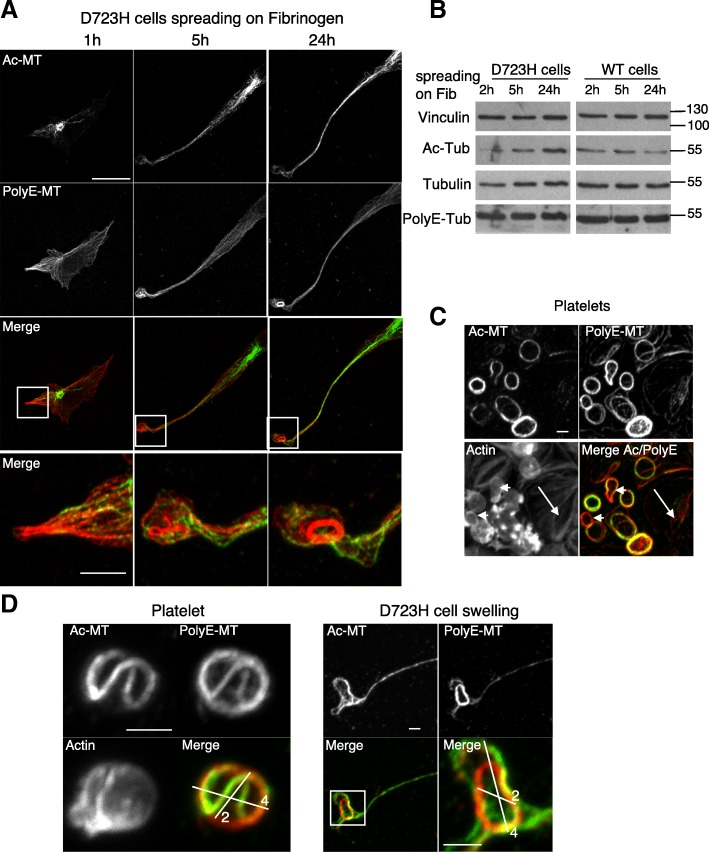
Specific subcellular localization of Ac-MTs and PolyE-MTs in PPLL and platelets. **a** Ac- and polyE-MT staining of D723H cells at different times (1, 5, and 24 h) of spreading on fibrinogen. Bars 20 μm. High magnification images of the merge signals are shown. Bar 5 μm. Ac- and PolyE-MT staining do not completely overlap. **b** Western blot of D723H and WT expressing CHO cell lysates successively probed with antibodies against Ac-Tub, PolyE-Tub, total tubulin, and vinculin; molecular weight markers in kilodalton are indicated on the right. **c** Images of actin staining, Ac-MT, and PolyE-MT decoration of the marginal band MT ring in purified platelets spreading on glass coverslips show that marginal band MT acetylation and polyglutamylation content are not perfectly overlapping. Bar 2 μm. **d** High magnification images of actin staining and Ac- and PolyE- decoration of marginal band MTs in a purified round platelet spreading on glass coverslips (left panels) and high and low magnification of MT bundles in a D723H swelling (right panels). Bars 2 μm. Ac-MT/PolyE-MT staining merges are shown with two cross bars of respectively 2 and 4 μm

As observed for megakaryocytes (Fig. 1a), in D723H cell spreading on fibrinogen the spatial localization of Ac- is different of PolyE-MTs (Fig. 2a). Indeed, Ac-MTs initially (1 h) surrounded centrosomes and later (5 h) concentrated along the elongating PPLL. In contrast, PolyE-MT staining was enhanced toward the PPLL distal end (1–5 h). After 24 h, long PPLL extensions were filled with Ac- and polyE-bundled MTs. In swellings at the tip of elongations, PolyE strongly stained MT rings, reminiscent of the platelet marginal band [7]. While starved cells or cells grown in the presence of serum show similar polyE- and Ac-MT content, resuspended cells prior fibrinogen spreading had lost most of these MT PTMs (Additional file 9: Figure S2). Most importantly, during the spreading kinetic of these resuspended D723H cells on fibrinogen, a progressive increase in Ac-MT content was observed cells while PolyE-MTs remained constant. This change in Ac-MTs was not observed in WT cells indicating that the upregulation of Ac-MTs may be important for sustained PPLL elongation (Fig. 2b).

In resting platelets, marginal band MTs are acetylated [26] but little is known about other MT PTMs. Platelets become activated while spreading on glass coverslips. We observed that marginal band MTs of resting, still round platelets stained both for Ac- and PolyE-MTs, but the modification patterns only partially overlapped (arrowheads) (Fig. 2c). In contrast, in fully activated spread platelets (arrows), polyE-MTs were lost, as has already been reported for Ac-MTs in which α-tubulin deacetylase HDAC6 mediates deacetylation [18]. Since MT coiling evolves during platelet activation [8, 27], we hypothesized that Ac- and PolyE-MTs could mark different platelet activation states. Indeed, differential PTM staining is best seen on a single human platelet with MT buckling (Fig. 2d, left) and resembles the PTM pattern of MTs in D723H swellings (Fig. 2d, right).

Taken together, our data show that acetylation and polyglutamylation marks on MTs of D723H cells elongating PPLL are distinct and dynamic. Moreover, D723H cells activate signaling pathways that induce swellings containing marginal band-like MT ring with similar size and PTM pattern to that of blood platelets.

### β1 tubulin is expressed in D723H cells. Behavior of highly polyglutamylated and β1 tubulin-containing MTs is similar in MKs and in D723H cells

Since proper platelet marginal band formation requires the expression of the megakaryocyte/platelet-specific β1tubulin isotype, we wondered whether β1tubulin could be expressed under α2bβ3 integrin signaling in D723H cells.

**Fig. 3:**
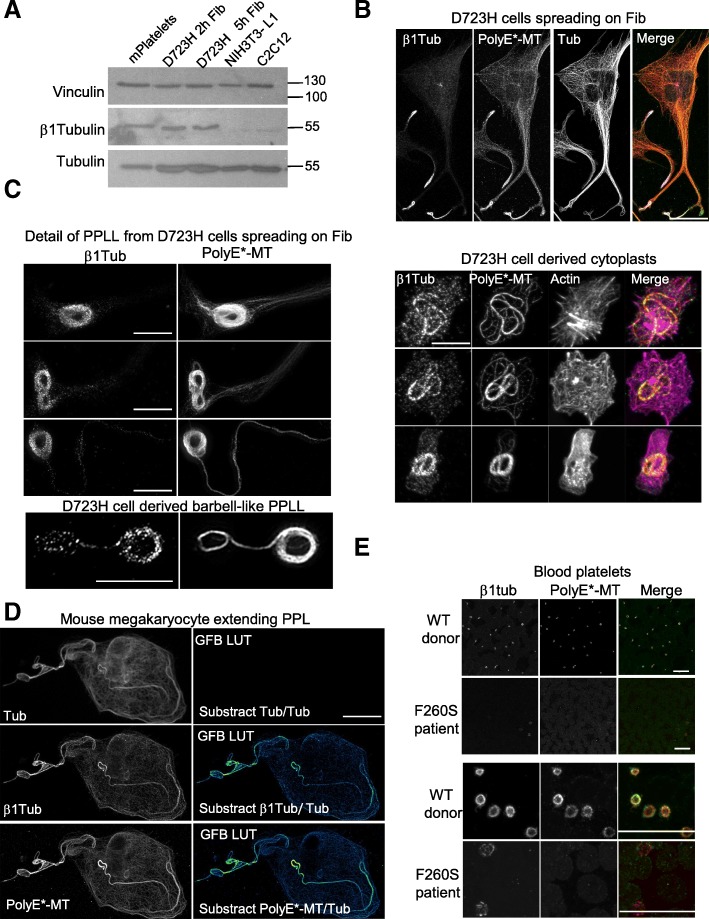
β1tubulin is expressed at low levels in D723H cells and is incorporated in polyE-rich MTs. **a** Western blot of platelets and cell extracts stained for indicated antibodies. β1tubulin is expressed in mouse platelets and in D723H cells spread for 2 and 5 h on fibrinogen but is not detectable in NIH3T3-L1 nor in C2C12 mouse cell lines. Alpha tubulin and vinculin are used as a loading control. Molecular weight markers in kilodalton are indicated on the right. **b** D723H cell spread on fibrinogen for 16 h and stained for indicated antibodies. Representative MIP images. β1tubulin**-**containing MTs concentrated in the far end of the PPLLs are polyglutamylated. Bar, 30 μm. **c** Left panels: representative examples of β1tubulin and PolyE colocalization at different stages of MT bundling in PPLLs and in an isolated barbell-like structure analyzed by STED microscopy. A single plane is shown. Bar 5 μm. Right panels: Representative examples of coiled MTs in cleaved cytoplasts stained for indicated antibodies. Cleavage is visualized by actin staining. Bar 10 μm. **d** MIP mature mouse megakaryocyte extending proplatelets stained with highly diluted antibodies for β1tubulin, polyE-MT, and total tubulin. Green fire blue LUTs (GFB LUT) was applied to substracted images. Polyglutamylated and β1tubulin-containing MTs are enriched toward PPL elongation tips. MIPs are shown. Bar, 12 μm. **e** Blood smears from healthy or β1tubulin F260S carrying patients were stained for β1tubulin and PolyE-MTs. Low magnification (top) and high magnifications (bottom) are shown. β1tubulin and PolyE-MTs are barely detectable in platelets with β1tubulin F260S mutation. Bars 20 μm

Using immunoblot, we observe that β1tubulin is expressed in platelets but also in D723H cells spreading on fibrinogen. In contrast, NIH3T3-L1 and C2C12 mouse cells display almost no reactivity with mouse β1 tubulin antibodies (Fig. 3a). Strikingly, β1tubulin-positive MTs, in D723H cells spreading on fibrinogen, concentrate in the dynamic protrusive end of PPLLs which are also highly polyglutamylated (Fig. 3b). Representative examples of β1tubulin and PolyE costaining (one Z section) at different stages of MT bundling in elongating PPLLs and in a severed barbell-like structure show that most β1tubulin pixels colocalize with PolyE-MTs (Fig. 3c left panels). We also present maximum intensity projection of D723H cells severed cytoplast structures visualized by actin staining (Fig. 3c right panels).

Expression of β1tubulin isotype is restricted to mature MKs extending PPL [12, 13]. Costaining of mouse liver-derived MKs with high dilution of polyE- and β1tubulin antibodies shows that, as we already observed in D723H cells, a gradient of β1tubulin-containing MTs exist in PPL of mouse MKs. This is better visualized by substracting total tubulin staining from respectively β1tubulin and polyE MTs staining (Fig. 3d). We then looked at PolyE stained and β1tubulin-containing MTs in human platelets. In healthy donors, platelet marginal band MTs which essentially contain β1tubulin, as a β isotype, are heavily polyglutamylated. Blood smears of patients with a heterozygous Tubb1 pF260S mutation contain only few platelets with strong MT disorganization, as reported [14]. In these patient platelets, we could not detect any polyglutamylation modification of these MT-like structures suggesting that a threshold of β1-tubulin-containing MTs must be attained to signal to polyglutamylases (Fig. 3e).

Our results show that in D723H cells, β1tubulin is expressed upon spreading on fibrinogen and locally enriched to the polyglutamylated part of the MTs that colonizes the extending PPLL. The potential link between β1tubulin expression and MT polyglutamylation is strengthened by our observations of mouse MKs. These original results further validate the D723H cell model to study microtubule PTMs during PPLL elongation.

### MT acetylation is required for PPLL extension

**Fig. 4:**
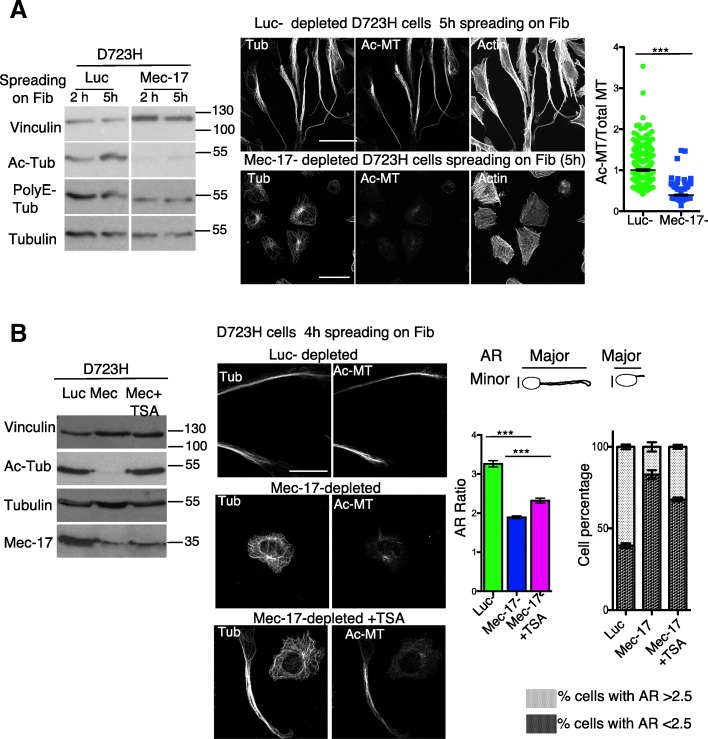
MT acetylation is required to induce PPLL extensions. **a** Luc- and Mec-17-depleted D723H cells were spread 48 h post siRNA (50 nM) on fibrinogen for 2 or 5 h. Cell extracts were analyzed by Western blot. Mec-17 depletion correlates with loss of Ac-Tub, while PolyE-Tub and total tubulin are not affected. Representative images (MIP) of luc- and Mec-17-depleted D723H cells stained as indicated. Quantification of Ac-MT/Total MT ratio shows the loss of Ac-Tub staining in MEC-17-depleted cells (scatter dot plot shows mean ± SEM; *n* = 3 at least 100 cells per condition were analyzed. Unpaired student *t* test, two-tailed ***P < 0.0001). Loss of Ac-Tubulin in Mec-17-depleted cells correlates with acquisition of a cell square shape phenotype. Bar is 30 μm. **b** D723H cells treated as in A, but using Mec-17 siRNA 15 nM. Carrier or TSA was added 30 min after cell spreading, and cells were analyzed 4 h later. Western blot shows that MEC-17 depletion is partial under these conditions. TSA treatment allows stabilization of Ac-MTs. MIP of representative images of luc-, Mec17, and Mec-17+TSA-depleted D723H cells stained for total and Ac-MTs. Bar is 20 μm. Rescue of PPLL elongation is quantified using the AR aspect ratio function (particle analysis) of ImageJ software that measures the ratio between the cell major and minor axis. (*n* = 3 at least 100 cells per condition were analyzed; graph, left panel: mean AR ratio, unpaired Student *t* test, two-tailed ***P < 0.0001; right panel graph: percentage of cells with AR ratio above or under 2.5 value, error bars are SEM

We first investigated the function of tubulin acetylation in PPLL extensions by siRNA of the acetyl transferase MEC-17 in D723H cells spreading on fibrinogen. MEC-17 depletion prevented MT acetylation, while levels of total and PolyE-MTs remained constant. Whereas control luciferase-depleted cells extended PPLLs containing Ac-MTs, MEC-17-depleted cells, which did not show acetylated MTs, remained square-shaped. This suggests that Ac-MTs are required for PPLL elongation (Fig. 4a).

To confirm this, we performed partial MEC-17 depletion and treated cells with trichostatin A (TSA), a pan-HDAC inhibitor [28]. Under partial MEC-17 depletion, MT acetylation was faintly detectable. TSA addition stabilized the remaining Ac-MTs, but did not change the steady state level of MEC-17 (Fig. 4b). Immunofluorescence showed that MEC-17-depleted cells in which Ac-MTs were not rescued by TSA treatment did not extend PPLL, whereas cells harboring Ac-MTs extended long protrusions. We quantified PPLL elongation by measuring the cell AR ratio (ratio of the cell major/minor axis). This confirmed the partial rescue of PPLL elongation by TSA-induced stabilization of Ac-MTs in MEC-17-depleted cells (Fig. 4b).

To study whether PPLL elongation only depends upon increasing MT acetylation, we treated WT cells with low doses of TSA, to mimic the Ac-MT level observed in D723H cells (Additional file 10: Figure S3A, left panel). TSA treatment induced cytoplasmic protrusions with several branches in WT cells (Additional file 10: Figure S3A, middle panel), but they did not elongate as much as in D723H cells, as quantified by AR ratio (Additional file 10: Figure S3A, right panel) and did not produce swellings at their ends.

Since lengthening of PPLL in D723H cells correlates with increased Ac-MTs, we wondered whether boosting the MT acetylation would result in longer PPLLs and/or more swellings. D723H cell spreading on fibrinogen were followed by live microscopy. After 2 h spreading, either TSA, the HDAC6-specific inhibitor tubastatin (TBSA), or vehicle was added to culture medium (00:00, Additional file 10: Figure S3B). In the presence of vehicle, PPLL continued to lengthen with time and swellings formed at their ends (Additional file 11: Movie S8). In contrast, TSA (Additional file 11: Movie S9) and TBSA (Additional file 11: Movie S10) treatment strongly inhibited PPLL lenghtening, as cytoplasmic branches widened over time and no swelling formation was observed. As TSA is toxic, cells were not imaged longer than 9 h after drug addition.

Next, we studied the importance of Ac-MTs in mouse liver-derived megakaryocytes elongating proplatelets. In vehicle-treated megakaryocytes, as expected, once proplatelet elongation is induced (07:30), many bead-like swellings are formed along the processes prior to platelet intermediates and platelets being released. The entire process takes several hours and results in megakaryocyte death (12:30) (Additional file 10: Figure S3C and Additional file 12: Movie S11). In contrast, addition of TBSA to megakaryocytes starting to elongate proplatelets (02:30) resulted in an arrest of the development of the proplatelet network which started to fuse to form stable giant lamellipodia (Additional file 10: Figure S3C and Additional file 12: Movie S12).

These data demonstrate that an increase in Ac-MT is required for efficient PPLL elongation in D723H cells, but is not sufficient to induce PPLL in WT cells. They also highlight the requirement for a regulation of the Ac-MT level both in D723H cells and in mouse MKs to protect the morphology of the elongating PPLLs.

### MT polyglutamylation is important to induce MT marginal band-like formation

The bundled MTs are heavily polyglutamylated in the swellings of PPLLs induced by αIIbβ3 D723H signaling, and in megakaryocyte-derived proplatelets (Figs. 1a, 2a, and 3b–d). To better investigate the function of MT polyglutamylation, we wished to increase the number of swellings formed, since D723H cells only produce in average one or two PPLL per cell.

Although the relationship between increasing megakaryocyte ploidy and platelet production is still a matter of debate [29, 30], we hypothesized that polyploidization of D723H cells could induce more processes. We thus used the Aurora-A inhibitor, MLN8237, that was further described to selectively inhibit Aurora B and induce polyploidy at higher concentration [31]. In preliminary experiments, we found that in D723H cells, MLN8237 concentrations above 200 nM induced cell endomitosis followed by polyploidization with no loss of cell viability. We thus treated D723H cells with 400 nM MLN8237 for 6 to 78 h. FACS analyses of DNA content showed that lenghtening exposure time to MLN8237 correlated with increased cell ploidy, with 16N being attained by 78 h treatment (Additional file 13: Figure S4A). Upon spreading on fibrinogen, polyploidization is accompanied by an increase in cell size and it correlates with increased numbers of PPLLs and more branching per PPLL, which results in the production of more swellings (Additional file 13: Figure S4B-S4C and Additional file 14: Movies S13-15). Spreading WT cells on fibrinogen or D723H cells on serum did not reproduce efficient PPLL elongation nor swelling formation (Additional file 13: Figure S4D and Additional file 14: Movies S16-18) further demonstrating the dependence of PPLL elongation for αIIbβ3D723H signaling. The MT network organization and MT PTMs in polyploid D723H cells (Additional file 13: Figure S4E) was similar to that of diploid cells (Fig. 2a).

**Fig. 5:**
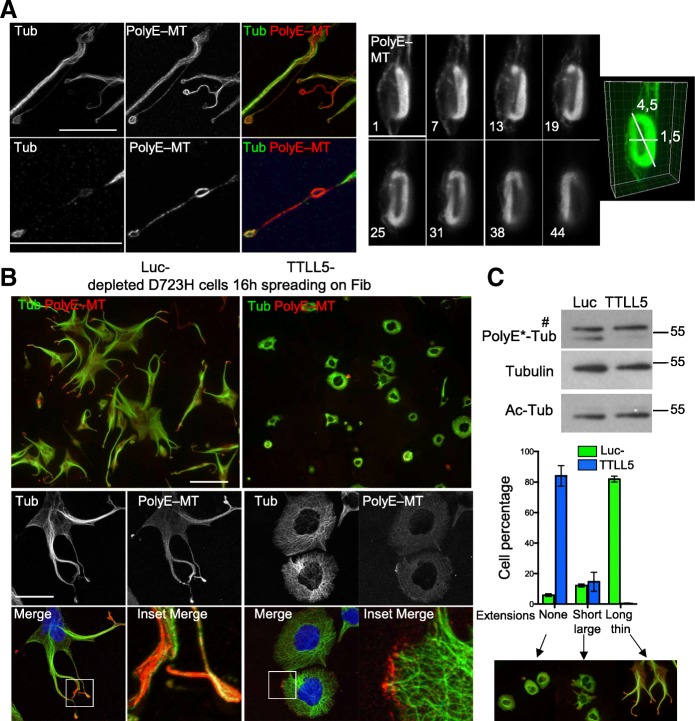
MT polyglutamylation is required for PPLL elongation. **a**–**c** Polyploid D723H cells. **a** Left panel: representative MIP image of PolyE and total MT staining in coiled MTs formed along a PPLL. Bar is 25 μm. Low (top) and higher (bottom) magnifications are shown. Ratio between intensities of PolyE decoration and total MTs varies. Right panel: numbered Z slices (from 45 images stack) and 3D reconstruction of PolyE-MTs decorating a MT marginal band-like structure, observed by STED microscopy. Bar is 5 μm. **b** Representative MIP images at low (top panels, bar is 125 μm) and high (lower panels, bar is 40 μm) magnification of Luc- and TTLL5-depleted cells and stained for total and polyE MTs. Merge and 20-μm merge insets are shown. Cells were treated for 24 h with MLN8237 (400 nM) then siRNA treated for 48 h (+MLN8237) and spread on fibrinogen for 16 h prior fixation. TTLL5 depletion prevents PPLL elongation. Remaining PolyE-MTs are localized at the edge of the cells. **c** Top panel: cell extracts were analyzed by Western blot. TTLL5 depletion correlates with loss of PolyE-Tub, while Ac-Tub and total tubulin are not affected. Number sign indicates that the band is non-specific (see the “Methods” section). Bottom panel: quantification of cell phenotypes following TTLL5 depletion. *n* = 3, at least 200 cells were manually counted for each experiment. Error bars are SEM

The intensity of PolyE-MT staining was elevated in swellings from MLN-treated D723H cells whereas total MT staining was not (Fig. 5a, left panels), indicating hyperglutamylation of these MTs. STED superresolution microscopy confirmed that the shape and volume occupied by marginal band-like PolyE-MT bundles in swellings are consistent with sizes reported for platelets derived from megakaryocytes (Fig. 5a, right panel). In MLN-treated D723H cells, depletion of TTLL5 polyglutamylase by siRNA removed most of the PolyE-MTs signal without affecting the Ac-tubulin signal (Fig. 5b, c). Loss of TTLL5 induced dramatic cell shape changes and an almost complete loss of extensions, as quantified (Fig. 5b, c). In the remaining short protrusions, MTs at the edges of the cells were always marked by leftover polyglutamylation, again indicating that this MT PTM is important to lead the PPLL elongation (Fig. 5b).

Our results show the requirement of MT polyglutamylation for efficient PPLL elongation in D723H cells. The striking localization of PolyE marks to the bundling MTs at the leading edge of the PPLL points out to novel functions of this MT PTM.

### β1tubulin expression and MT polyglutamylation depend upon each other for efficient MT bundling and coiling during PPLL elongation

**Fig. 6:**
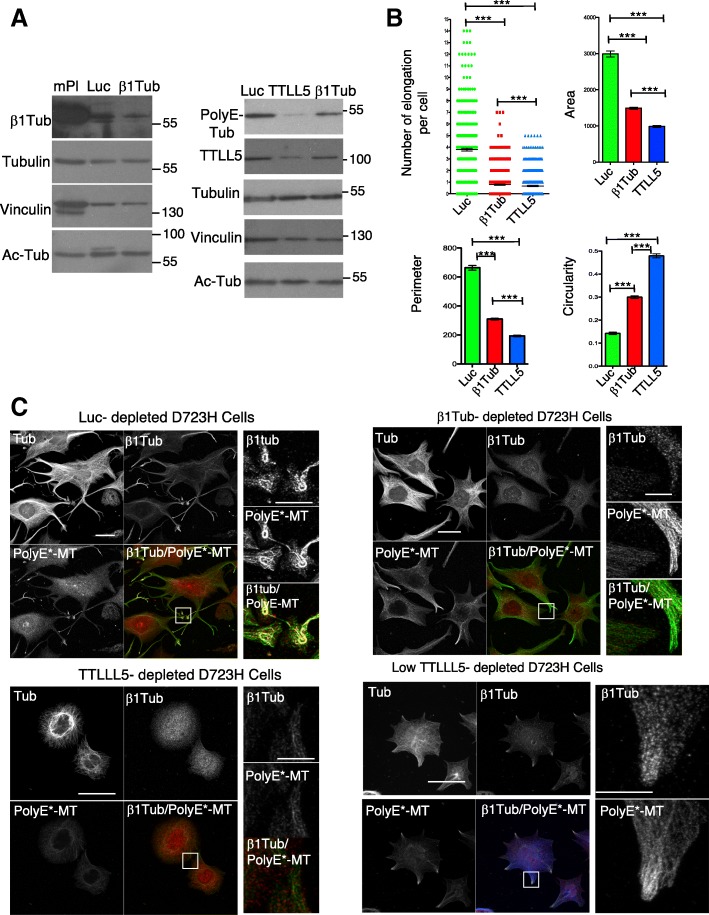
β1tubulin-containing MTs regulate PolyE-MT fasciculation and coiling in PPLL. **a** Total protein extracts of mouse platelets and Luc-, β1tubulin-, and TTLL5-depleted polyploid D723H cells analyzed for indicated antibodies. β1tubulin and TTLL5 are partially depleted following siRNA treatment, and depletions do not affect Ac-MTs levels. β1tubulin depletion does not affect polyE-MTs while TTLL5 does. **b**, **c** Luc-, β1tubulin, and TTLL5-depleted polyploid D723H cells stained for total tubulin, βtubulin, and PolyE-MTs after 16 h spreading on fibrinogen. **b** Quantification of number of elongations per cell and several shape descriptors using FIJI software (*n* = 3, at least 150 cells were analyzed per condition). Error bars are SEM (*p* values < 0.0001, two-tailed *t* test for each test). **c** Representative images of luc-, β1tubulin-, TTLL5, and low TTLL5-depleted cells stained for tubulin β1tubulin and PolyE-MTs (bars, 50 μm and 10 μm for high magnification insets)

The importance of the β1tubulin isotype in proper MT marginal band formation in platelets is well known, but the mechanisms involved are not understood. We found that a low level of β1tubulin expression is correlated with high MT polyglutamylation (Fig. 3). To better understand if there exists a relationship between β1tubulin expression and polyglutamylation, we depleted β1tubulin and TTLL5 by siRNA in polyploid D723H cells. β1tubulin was only partially depleted in D723H cells, and its depletion did not affect total tubulin nor acetyl-tubulin levels (Fig. 6a, left; Additional file 15: Figure S5A). While TTLL5 depletion affected the polyglutamylase expression, and consequently PolyE-MT levels, depletion of β1tubulin did not (Fig. 6a, right). As shown before, control depleted cells extend long PPLLs with at their ends, swellings that contain highly coiled β1tubulin-containing PolyE-MTs. In contrast, such swellings did not form upon loss of β1tubulin. Cytoplasmic elongations that extend from the cell body remained larger and were filled with PolyE-MTs, but extreme fasciculation of these PolyE-MTs did not occur (Fig. 6c and Additional file 15: Figure S5B). As observed earlier (Fig. 5b), loss of TTLL5 essentially prevented elongation of cytoplasmic branches and β1tubulin staining remained diffuse, as if the tubulin isotype was not properly incorporated into MTs (Fig. 6c). The effects of knockdowns on cell shape were quantified using different parameters. The circularity changes reflect that in β1tubulin, unlike in TTLL5-depleted cells, PolyE-MTs induce cytoplasmic elongations, but perimeter and area values show that they do not extend far (Fig. 6b). The correlation between MT polyglutamylation and β1tubulin-containing MTs was confirmed in TTLL5-depleted cells with residual polyE-MTs. In these star-like cells, the low level of PolyE-MTs was systematically concentrated at the leading edges of the small protrusions and had incorporated the β1tubulin isotype (Fig. 6c, TTLL5 low).

To analyze whether β1tubulin-containing MTs could be better targets for MT polyglutamylases, we overexpressed alone or in combination, gfp fusion of αtubulin, β1tubulin, and common β4 tubulin isotypes, in D723H cells. We did not detect, in immunofluorescence, increased polyglutamylation of β1tubulin-containing MTs (Additional file 15: Figure S5C). This could reflect the poor incorporation of gfp-β1tubulin in MTs. Alternately, β1tubulin targeting to pioneer MTs and/or its coexpression with a specific and yet unknown αtubulin isotype might be required for effective alpha or beta tubulin polyglutamylation of MTs.

We show that β1tubulin expression is mandatory for PPLL elongation. β1tubulin does not regulate the polyglutamylation level of MTs, but PolyE-MTs devoid of β1tubulin isotype are no longer capable of bundling nor coiling. We provide the first insight that the intimacy between β1tubulin-containing MTs and polyglutamylation is required for proper MT fasciculation and coiling required for platelet release.

## Discussion

Previous elegant works highlighted the importance of MT dynamics and dynein-dependent MT sliding for proplatelet elongation [5, 32]. Knock-out mice models and cultured megakaryocyte studies implicated Rac/Cdc42 GTPases and their p21-activated kinase (PAK) effector, PKC substrate MARCKS, RhoA, and its effector DIAPH1 [33–37] in the regulation of MT and/or actin networks. Moreover, mutations in cytoskeleton-associated proteins, such as FLNA, ACTN1, MYH9, or TUBB1, were identified in patients affected by proplatelet defects and thrombocytopenia [15, 38–40].

However, due to difficulties in manipulating megakaryocytes and lack of models to modulate cytoskeleton elements throughout proplatelet elongation, these pathways remain poorly understood.

The platelet membrane GPIb-IX-V complex is important for proplatelet formation [20], but engagement of αIIbβ3 integrin on fibrinogen is sufficient to initiate proplatelet formation from mouse megakaryocytes [21]. Expression in CHO cells of a constitutive but partially activated αIIbβ3 integrin (D723H cells) was shown to promote elongation of MT-dependent cytoplasmic branches [22, 23]. Here, we characterized D723H cells. We demonstrate that engagement of αIIbβ3D723H integrin to fibrinogen is sufficient to recapitulate MT behavior previously described in cultured megakaryocytes elongating proplatelet [19, 41]. In that regard, D723H cell is a unique tool to study the regulation of MT rearrangements.

Fibrinogen-engaged D723H cells elongating proplatelets are filled with dynamic MTs that bundle toward the tip of the elongation. Electron microscopy, 3D SIM, and STED microscopy approaches show that MT coiling occurs in the terminal swellings and that cytoplasts are released in cell culture medium. In mature polyploid megakaryocytes, recruitment of the demarcation membrane system (DMS) [42] together with actin signaling allows the elongation and branching of the proplatelet [5] and results in the formation of multiple swellings from a single megakaryocyte. To overcome the limitation of diploid D723H cells, which promote elongation of only one or two PPLL, we induced D723H cell polyploidization. Strikingly, polyploidization not only increased the number of PPLLs but also their branching activity, resulting in the production of multiple swellings per cell, probably as a result of a bigger protein/lipid reservoir. However, polyploid D723H cells never produced cytoplasts in amount similar to the estimated 4000 platelets produced by one mature megakaryocyte. We did not investigate membrane remodeling or actin dynamics in polyploid D723H cells, but since cytoplasmic accumulation of mRNAs/proteins promotes PPLL branching, we believe the activation of the actin machinery by D723H integrin signaling is likely. Importantly, severed cytoplasts were observed throughout the cell culture medium, which demonstrates that proteins necessary to the PPLL shaft thinning are expressed before severing occurrence.

We observed that PPL-MTs from fixed mouse megakaryocytes are extensively modified by both acetylation and polyglutamylation with discrete differences in the PTM pattern of MTs. Using the D723H cell model, we found that acetylation and polyglutamylation differently mark the PPLL MTs. MT acetylation occurred along the MTs colonizing the extending PPLL, and its steady state level increased with elongation kinetics likely because more “MT substrates” become available. MT acetylation is required for PPLL elongation and must be regulated since excessive acetylation induces PPLL widening and prevents swelling formation*.* Thus, tight control of the balance between MT-acetylating and deacetylating enzymes must occur for efficient PPLL elongation. Interestingly, MT acetylation mediates dynein-dependent transport of mitochondria during inflammasome activation [43] and enhances dynein binding in vitro and in vivo [44]. It would thus be of interest, in future studies, to study whether Ac-MTs mediate dynein recruitment on MTs and consequently the dynein-dependent MT sliding mechanism required for PPLL elongation [5].

In the D723H cell model, we observed polyglutamylation of MTs in the most dynamic growing region of the PPLL, in the swellings and in severed cytoplasts in which coiling and even buckling occurred. Fibrinogen engagement to αIIbβ3D723H integrin in CHO cells is sufficient to recapitulate specific hematopietic features such as the remarkable MT marginal band structures described in preplatelets, barbell platelets, and platelets. In the megakaryocyte lineage, these MT structures depend upon the expression of mature megakaryocyte-specific β1tubulin isotype [13], although why this is the case is unknown. Curiously, β1tubulin is expressed when mature megakaryocytes start to elongate proplatelets [12] indicating that earlier expression might be toxic and suggesting a specific role of this isotype in proplatelet extension. We found that β1tubulin is actually expressed in D723H cells. Interestingly, the C-terminal tail of β1tubulin is the most divergent sequence from all other tubulin isotypes. It is noteworthy that this completely unstructured peptide is the target of polyglutamylases in all tubulin isotypes.

During PPLL elongation, we show a strong correlation between localized enrichment of polyglutamylation and β1tubulin incorporation in MTs. Loss of β1tubulin prevented a good fasciculation of the polyE-MTs in the far end of the elongating PPLL while not affecting their steady state level. However, ectopic expression of β1tubulin-gfp failed to prove that its C-terminal tail contains specific signals to attract polyglutamylases. We, therefore, are currently investigating whether β1tubulin targeting to the dynamic MTs colonizing the far end of the PPLL may be required for signaling to polyglutamylases. Moreover, while we show that polyglutamylation of β1tubulin-containing MTs is required for platelet formation, we could not determine whether it is β1tubulin itself or its associated specific α subunit heterodimer partner that is a target of polyglutamylation. The fact that depletion of TTLL5, an enzyme shown to have a preference for alpha tubulins in mammalian cells [45], removes the polyglutamylation of β1tubulin-containing MTs opens the possibility that β1tubulin recruits and/or stabilizes the polyglutamylase on the MT as it modifies the neighboring α tubulin subunit.

Valenstein and Roll-Mecak [46] showed, using purified tubulins, that polymerized MTs with defined levels of glutamate residues recruit and activate the MT-severing enzyme spastin. Spastin recruitment/affinity increases with length of the glutamate chains, but its activity peaks before being inhibited by a biphasic mechanism. Long polyE chain-MTs loaded with spastin in a non-productive binding mode induce MT stabilization [46]. These findings explain earlier observations of MT cross-linking activity of inactive spastin mutants [47, 48]. Our observations of MT behavior during PPLL elongation fit nicely with this model. At the beginning of PPLL elongation, MTs are highly acetylated and MT severing is likely to occur to promote elongation by dynein-dependent MT sliding. Severing might require Ac-MT-dependent induction of katanin severing enzyme [49] or spastin activation by short polyE chain-containing MTs. In contrast, when PPLLs are long enough, the PPLL shaft becomes thinner and contains extremely fasciculated and highly polyglutamylated-β1tubulin-containing MTs, which could be a reservoir for inactive spastin protein. It is indeed difficult to assess the length of PolyE chains on tubulins in vivo, and more work will be needed to confirm this model. Although we did not identify the mechanism of polyglutamylase recruitment to β1tubulin-containing MTs, we propose these β1tubulin-MTs could tune polyglutamylation and consequently spastin activity. This would be consistent with the extensive damage of the MT network induced by overexpression of β1tubulin in CHO cells [50].

The marginal band MTs present in platelets maintain their flat discoid morphology [7]. Modeling and in vitro approaches showed that without any MT motors, MTs spontaneously organize and coil longitudinally along the wall of elongated microchambers [51] and that cortical tension is required together with marginal band MT stiffness to determine the platelet size [9]. We observed that during platelet activation, buckled marginal bands are differently modified by MT PTMs. It will be interesting to address MT PTM functions in platelet size and shape evolution by modeling approaches.

Among platelet disorders, Glanzmann thrombastenia (GT; dysfunction of αIIbβ3 integrin) and Bernard Soulier syndrome (BBS; dysfunction of GPIb-IX-V complex) affect platelet surface glycoproteins. Mutations in these receptors, which mediate platelet aggregation, induce mild to extremely severe patient bleeding. In addition, these receptors are also involved in proplatelet formation and the reorganization of MT cytoskeleton [52, 53]. Remarkably, in a BBS mouse model, fewer but larger platelets contain increased number of microtubule coils in an oversized microtubule marginal band [53]. Changes in the MT content of platelet marginal band was also reported in other giant platelet disorders such as MYH9-related disorders or Gray platelet syndrome [54, 55]. But today, these observations remain poorly documented and are still not understood. Indeed, these defects in MT coil size and coiling efficiency derive from mutations in completely unrelated genes inducing different signaling cascades. In this context, it would be of interest to study the correlation between MT acetylation/polyglutamylation and MT coiling and bundling in the giant platelets of patients affected by these different disorders.

## Conclusions

In this study, we established and validated a convenient cell model that recapitulates the MT behavior observed in megakaryocytes elongating proplatelets from the bone marrow through the blood sinusoids. We demonstrate that these highly specific hematopoietic features can be phenocopied by αIIbβ3 integrin engagement on fibrinogen independently of any other hematopoietic signaling. Our results reveal the importance of MT acetylation and polyglutamylation for efficient rearrangements of MTs during proplatelet elongation and platelet release. We provide the first insights of how the elusive hematopoietic β1tubulin isotype could function in microtubule coiling.

Our study more broadly suggests that temporal expression of tubulin isotypes in differentiating specialized cells could signal specific MT post-translational modifications and acquisition of specific MT behavior.

## Methods

### Cell lines

CHO cells expressing integrin αIIbβ3WT or D723H were a kind gift of Dr. Kieffer; both cell lines were previously described [23]. αIIbβ3 integrin expression at the cell surface was periodically verified by incubating live cells with A2A9 antibody (Santa Cruz Biotechnology Cat# sc-21783, RRID:AB_627817), a conformational and blocking antibody that recognizes the native integrin complex, prior fixation, and immunofluorescence (C2C12 mouse myoblast ATCC Cat# CRL-1772, RRID:CVCL_0188, 3T3-L1 mouse fibroblast (ATCC Cat# CCL-92.1, RRID:CVCL_0123)). All cell lines were grown according to the standard protocols. All cell lines were routinely tested for the absence of mycoplasma contamination.

### Megakaryocytes

The livers from E13.5 pregnant OF1 female mouse embryos were placed in RPMI medium. Megakaryocytes were differentiated for 4 days from murine E13.5 fetal liver cells with 50 ng/ml TPO [56]. Animal experiments were conducted according to procedures approved by the local (agreement number 34-366 for B.B.B.) and Regional Ethics committees.

### Platelets

Nontherapeutic buffy-coat diluted in PBS was centrifuged (10 min, 400*g*), platelet-rich plasma was collected, and platelets (10^6^/well, 24-well plate) were spun 3 min (600 g) on glass coverslips and fixed. For peripheral blood smears of a woman with TUBB1 p.F260S mutation, written informed consent was obtained from her in accordance with the Declaration of Helsinski. The Institutional Review boards of Nagoya Medical Center and Miyagi Chidren’s Hospital approved the study [14].

### Antibodies and reagents

Rabbit antibodies against alpha tubulin C102 was a gift of M. Andreu [57]. Mouse β1tubulin was raised against its C-terminal 19 aa peptide [15].

The polyclonal polyglutamylated tubulin antibody (polyE-tub) was developed against Cys (Glu)_9_ peptide coupled to keyhole limpet hemocyanin [45] and was first described by M Gorovsky [58]; it only recognizes long glutamate side chains (> 3 glutamates). The monoclonal glutamylated tubulin antibody GT335 (PolyE*-Tub, (AdipoGen Cat# AG-20B-0020, RRID:AB_2490210, batch A27791601) recognizes the branching site where glutamate residues are added and react with both short and long glutamate side chains. It also reacts with polyglutamylated nucleosome assembly protein 1 (NAP1) that is nuclear and gives a signal just above the one of polyglutamylated tubulin in Western blot. Depending on other antibodies used in the experiments, either polyE or PolyE* were used. Both antibodies stained the same MT bundles and coils, in all described experiments.

Rat antibody against tubulin (YOL, sc-53030, batch I1912) and monoclonal antibodies against Ac-Tub (6-11B-1) (Santa Cruz Biotechnology Cat# sc-23950 RRID:AB_628409, batch C0317), tubulin (DM1-A, Sigma-Aldrich Cat# T9026, RRID:AB_477593, batch 083M4847V) and vinculin (Sigma-Aldrich Cat# V9131, RRID:AB_477629, batch 018M4779V) were used.

The following reagents were obtained from different companies: fibrinogen (Enzyme Research, IN, USA), mouse thrombopoietin (GIBCO), tubastatinA hydrochloride (MedChem Express), trichostatin (SIGMA), MLN8237 (SelleckChem), and SiR-tubulin (spirochrome, Cytoskeleton Inc).

### siRNAs, plasmids, SiR tubulin, and transfection

We used Eurogentec-synthesized siRNA to deplete endogenous mRNA from CHO cells. Cricetulus targeting sequences were as follows: for luciferase, 5′ CGUACGCGGAAUACUUCG(dT)(dT)3′; for Mec-17, 5′ CCACACCAACUGGCCAUUGA (dT)(dT)3′; for TTLL5, two oligo sequences were mixed, 5′ CAGCAACAGGCCACAGA(dT)(dT)3′ and 5′ CAGGCGGAACCCUUUUCAAAG(dT)(dT)3′; and for TUBB1, 5′ AACAAGAUCAGGGAGGAA U (dT)(dT)3′.

Plasmid sfGFP-EB3-7 (Addgene plasmid # 56481) and Plasmid mCherry-Lifeact-7 (Addgene plasmid # 54491) were gifts from Michael Davidson.

siRNA/plasmids were transfected for 48/24 h using Lipofectamine/RNAiMAX or Lipofectamine/plus reagents (Invitrogen). CHO cells were serum starved (2 h), washed and seeded on fibrinogen-coated (50 μg mL^−1^) glass coverslips. Drugs were added 30 min to 2 h after seeding. For MLN8237 (400 nM)-induced polyploidization, cells were treated 24–48 h prior to transfection and until the end of the experiment. SiR Tubulin was applied on cells overnight at 80 nM with 2 μM verapamil, the day before spreading cells on fibrinogen.

### Cell fixation, immunofluorescence microscopy, live cell imaging, quantification

Cells and megakaryocytes were fixed with 4% paraformaldehyde in PEM (0.1 mM PIPES, pH 6.9; 1 mM EGTA; 0.5 mM MgCl2) containing 0.2% Triton X-100 for 10 min at 37 °C or in MeOH for 10 min at − 20 °C, blocked with 1% BSA and stained with appropriate antibodies diluted in PBS containing 1% BSA for 1 h, then washed five times in PBS and incubated with Alexa 350-, 488-, and 555-conjugated secondary antibodies (Life Science) and when indicated with Alexa 647-conjugated phalloidin (to visualize actin staining). Cells were mounted in Mowiol with anti-fading N-propyl gallate.

Fixed cells were observed with wide field or confocal microscope as indicated.

Wide field fluorescence microscopy was performed using a Zeiss AxioimagerZ1 microscope. Mosaic images were acquired with a Zeiss 40x EC Plan-NEOFLUAR 1.3 oil DIC or Zeiss 20× Plan Apochromat 0.8 and an Andor Zyla 4.2 sCMOS camera. To increase sampling size, 20 to 50 fields of each experimental condition were analyzed. Images were segmented using an intensity threshold in ImageJ software. Unresolved clusters of cells were discarded. Cell Ac-Tub and total tubulin staining mean intensities were calculated, and cell elongation was monitored using the shape descriptors such as aspect ratio (AR; ratio of major to minor cell axis lengths), cell perimeter, and area. Values were entered in a prism software to calculate Ac-Tub/tubulin ratio, AR and perimeter, SEM and *p* values.

Confocal microscopy was performed using a Leica SP8 confocal microscope equipped with a Leica 63x HCX PL APO 1.4 oil CS2 objective. Most images are maximal intensity projections (MIP) of 3D Z stacks. Sometimes, single planes are shown as indicated.

For modulation contrast time-lapse microscopy, images were recorded using either an inverted Leica DMIRE2 microscope, a 20X N PLAN L 0.4 LMC (Leica Modulation Contrast) objective and a Princeton Instruments Micromax YHS1300 CCD camera or an Olympus IX83 microscope, a 20x LUCPLFLN 0.45 RC2 (Olympus Relief Contrast) and an Andor Zyla 4.2 sCMOS camera. Both systems were driven by metamorph software.

For cytoplast fluorescence imaging (Fig. 1c), mCherry-Lifeact and GFP-EB3 comets in D723H cell-derived cytoplasts were acquired with a Delta vision Personal PDV microscope equipped with an Olympus 60x PlanApoN 1.42 oil objective and a Photometrics CoolSNAPHQ2 CCD camera.

High-speed widefield fluorescence imaging (including widefield FRAP experiments) was performed using a Deltavision OMX microscope equipped with a 60x Plan Apochromat 1.3 Sil objective and Photometrics Evolve 512B EMCCD cameras for each channel and 488-, 561-, and 642-nm LASER excitation. 3D stacks of five to seven planes were acquired every second. Images were further processed using the Huygens deconvolution software (Scientific Volume Imaging) and a qMLE algorithm. Unless specified, maximum intensity Z projections are displayed.

STED superresolution microscopy was performed using StarRed and STAR635P-coupled secondary antibodies (Abberior) and an Abberior Instruments Expert Line STED microscope (Olympus IX83 microscope stand and 100X UPLSAPO 1.4oil lens, QUADScanner, 561- and 640-nm pulsed excitation LASERs and a 775-nm pulsed depletion LASER. 3D STED stacks were acquired using the easy3D SLM module.

### Electron microscopy

D723H cells spread on fibrinogen-coated cover slips were prepared for scanning electron microscopy to study their surface by fixation for an hour in 2,5% glutaraldehyde in PHEM buffer, pH 7.2 for an hour at room temperature. To examine cytoskeletons, cells were permeabilized as described in [6] prior to fixation, then washed in PHEM buffer. Fixed samples were dehydrated using a graded ethanol series (30–100%), followed by 10 min in graded ethanol–hexamethyldisilazane, and then hexamethyldisilazane alone. Subsequently, the samples were sputter coated with an approximative 10-nm-thick gold film and then examined under a scanning electron microscope (Hitachi S4000, at CoMET, MRI-RIO Imaging, Biocampus, INM Montpellier France) using a lens detector with an acceleration voltage of 10 KV at calibrated magnifications.

For transmission electron microscopy, cells were fixed for an hour in 2,5% glutaraldehyde in a PHEM buffer (1X, pH 7.2) overnight at 4 °C and post-fixed in a 0.5% osmic acid for 2 h at dark and room temperature, dehydrated in a graded series of ethanol solutions (30–100%), and embedded in EmBed 812 using an Automated Microwave Tissue Processor for Electronic Microscopy, Leica EM AMW. Thin sections (70 nm; Leica-Reichert Ultracut E) collected at different levels of each block were counterstained with uranyl acetate 1.5% in 70% ethanol and lead citrate and observed using a Tecnai F20 transmission electron microscope at 200 KV in the CoMET MRI facilities, INM, Montpellier France.

### Statistical analysis

All experiments described in the manuscript were at least performed three times unless otherwise mentioned. Data were analyzed with Prism5 for MacOS X. Data were analyzed using unpaired, two-tailed *t* tests. ***P < 0.0001 was considered statistically significant. The values are presented as the means, and error bars represent the standard error of the mean (SEM). Raw data are provided in Additional file 16.

## Additional files

Additional file 1: **Figure S1.** D723H cells elongate PPLL terminated by swellings containing bundled and coiled MTs. (A). Modulation contrast time lapse of the formation of PPLLs after spreading on fibrinogen (*t* = 00:00:00). PPLLs form in D723H but not in WT cells. Bar 45 μm. (B) Low and high magnification of MT bundles coiling in a D723H derived swelling. Representative images shown were acquired by scanning electron microscopy of extracted D723H cells spread on fibrinogen. Bars 2.5 μm. (C-E) High speed wide field fluorescence imaging. (C) mCherry-Lifeact and GFP-EB3 comets in D723H cell-derived cytoplast were acquired every 5 min. A single slice is shown at different time points as indicated. Bar is 10 μm (D) Images of the behavior of coiled MTs with fluorescent SiR-Tubulin in a D723H dependent swelling is shown at indicated times. Bar is 7 μm. (E) low and high magnification images of GFP-EB3 comets in a D723H cell-derived barbell platelet acquired every second, MIP of 5 planes is shown. Bar is 10 μm. (PDF 3106 kb)

Additional file 9: **Figure S2.** Pattern of Ac-and PolyE-MTs in D723H cells. Western blot of D723H CHO cell lysates. Lysates were prepared from adherent cells grown in presence of 10% fetal calf serum (Ser+) or serum starved for two hours (Ser-), from cells in suspension after two hours serum starvation (Susp), from suspended cells spread on fibrinogen for 2 h (Fib 2 h) or 24 h (Fib 24 h). Lysates were successively probed with antibodies against Ac-Tub, PolyE-Tub, total tubulin and vinculin as a loading control. Molecular weight markers in kilodaltons are indicated on the right. (PDF 83 kb)

Additional file 10: **Figure S3.** Increasing MT acetylation in WT cells is not sufficient to induce PPLL elongations. (A) WT cells treated with low TSA drug concentrations (ng/ml) and spread for 16 h on fibrinogen were analyzed for increased total acetylation level by western blot and for induction of PPLL extensions by immunofluorescence. Representative low magnification and wide field acquired tubulin stained images are shown to visualize cell shape (Bar, 150 μm). Quantification of the AR ratio: *n* = 3, at least 200 cells per condition were analyzed. Graph shows mean AR ratio, unpaired student t-test, Two tailed ***P < 0.0001. Error bars are SEM (B-C) Modulation contrast time lapse. Cell edges were enhanced using the ImageJ software process enhance contrast function (8%). (B) D723H cells treated with carrier, TBSA (10 μM) or TSA (100 ng/ml) were analyzed for increased Ac-MTs level by Western blot (6 h drug treatment) and for PPLL behavior by time lapse. Bar 50 μm. In drug treated cells, induced PPLL tend to retract and widen with time. (C) Mouse liver-derived-megakaryocytes spread on fibrinogen are treated with carrier or TBSA. Bar 80 μm. As observed on D723H cells, TBSA treatment induced retraction and widening of induced proplatelet (*n* = 2, 15 megakaryocytes). For better visualization please see corresponding movies. (PDF 9633 kb)

Additional file 11: **Movie S8-S10.** MT acetylation level must be controlled for efficient PPLL formation in D723H cell spreading on fibrinogen. D723H cells spreading on fibrinogen treated with carrier, TSA or TBSA as indicated and imaged every 5 min with an inverted Leica DMIRE2 microscope equipped with a Leica 20X N PLAN L 0.4 LMC and driven by metamorph software. (ZIP 2318 kb)

Additional file 12: **Movies S11.** and **S12.** MT acetylation level must be controlled for efficient proplatelet formation in mouse megakaryocyte. Megakaryocytes extending proplatelets on fibrinogen treated with carrier or TBSA as indicated. Modulation contrast time-lapse microscopy images were acquired every 15 min. (ZIP 2690 kb)

Additional file 13: **Figure S4.** D723H polyploidization increases number and branching of PPLLs when spread on fibrinogen. (A-C) D723H cells treated with MLN8237 (400 nM) for indicated times. Cells were analyzed for DNA content by flow cytometry (A) or spread on fibrinogen for 4 h before analyzing their behavior by modulation contrast time-lapse microscopy. (B, bar is 30 μm). (C) Quantification of PPLL numbers and branching in D723H polyploid cells treated as in (A-B) as a function of duration of MLN treatment and spread for 16 h on fibrinogen. MLN8237 treatment increases D723H cells ploidy and their capacity to extend more PPLLs bearing more branchings and more swellings. n = 3 at least 50 cells per condition were analyzed, error bars are SEM. (D) D723H or WT cells treated with MLN8237 (400 nM) for 72 h before spreading on fibrinogen or cultured with serum as indicated, and analyzed by modulation contrast time-lapse microscopy. Nor WT cells spread on fibrinogen nor D723H cells cultured with serum do produce PPLLs, a phenotype observed only in D723H cells spread on fibrinogen. Bar is 50 μm. (E) Representative MIP images of polyploid D723H cells spread on fibrinogen for 16 h and stained with indicated antibodies. Polyploidization does not modify Ac-, polyE- nor β1 MTs localization in PPLLs. Bar 60 μm. (PDF 4828 kb)

Additional file 14: **Movies S13–S18.** Polyploidization increases PPLL and swelling formation in D723H cells spreading on fibrinogen but has no effect on WT cells spreading on fibrinogen or D723H cells in serum. D723H cells spreading on fibrinogen treated as indicated. Modulation contrast time-lapse microscopy images were acquired every 5 min. (ZIP 13932 kb)

Additional file 15: **Figure S5.** Ac-MTs distribution is not affected by loss of β1tubulin or TTLL5. (A) Luc-, β1tubulin**-** and TTLL5- depleted polyploid D723H cells stained for total MTs, Ac-MTs and PolyE-MTs after 16 h spreading on fibrinogen. Bar is 100 μm. (B) Low magnification of representative merge images of Luc- and β1tubulin- depleted D723H cells stained for total MTs (red) and PolyE-MTs (green) show the cell shape change in β1tubulin depleted cells. Bar is 160 μm. (C) MLN treated and Gfp-α tubulin (αtub) or tubulin β4-Gfp (β4tub) or tubulin β1-Gfp (β1tub) or αtub plus β4tub or αtub plus β1tub transfected D723H cells were analyzed for Gfp expression, total tubulin and PolyE-MTs stainings. Representative images (MIP) are shown. Over 300 overexpressing cells were monitored for each condition. None showed a marked increase in PolyE-MTs staining. Bar 70 μm. (PDF 6370 kb)

Additional file 16: Raw Data values. (XLSX 267 kb)
