# Supplementary figures and images for: Microtubule polyglutamylation and acetylation drive microtubule dynamics critical for platelet formation
Source: BMC Biol. 2018 Oct 18;16:116. doi: 10.1186/s12915-018-0584-6 (PMC6194603; doi:10.1186/s12915-018-0584-6)

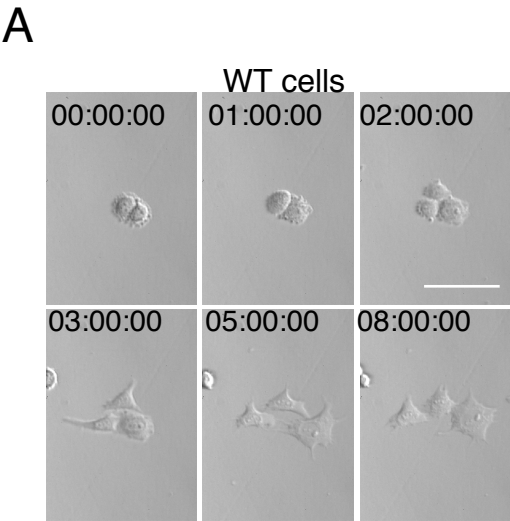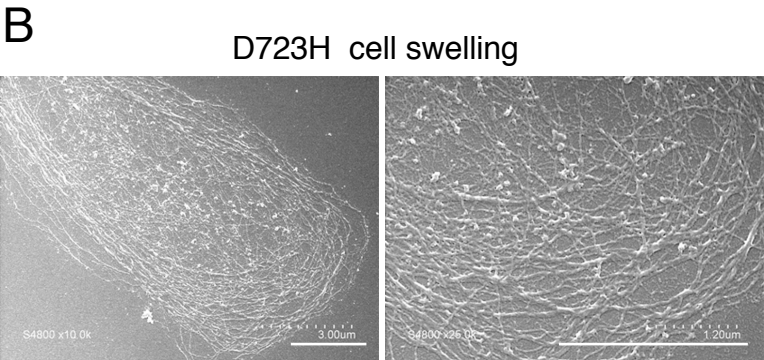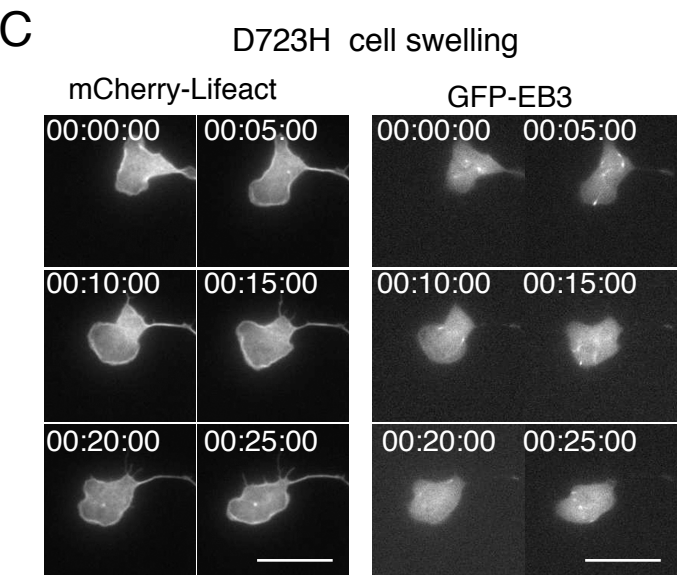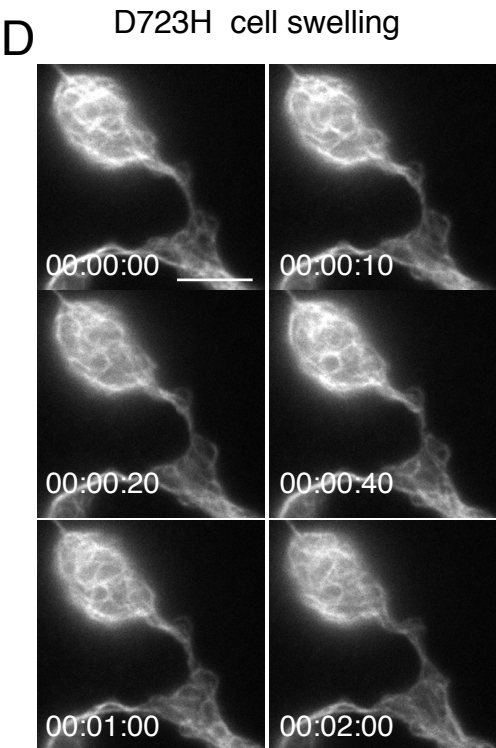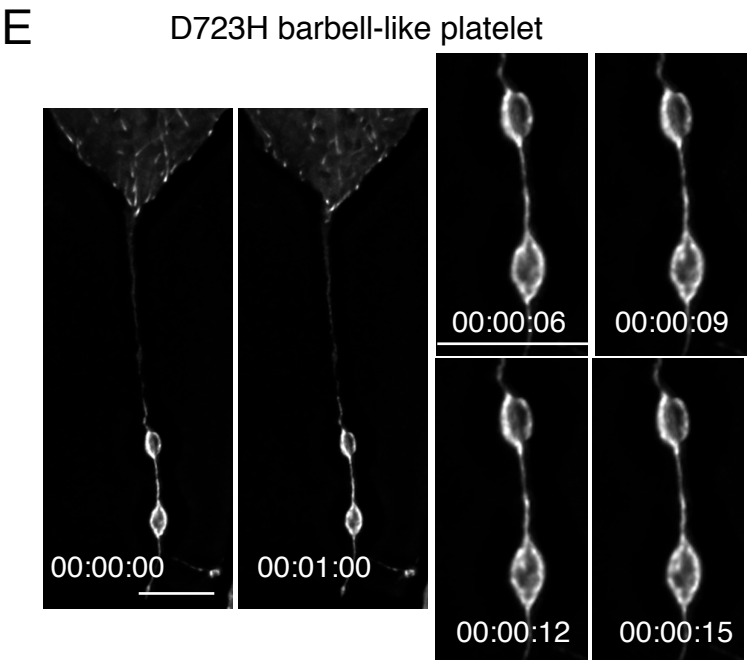

Suppl. Figure 1

Supplement: Supplementary file 8 — Figure S1. D723H cells elongate PPLL terminated by swellings containing bundled and coiled MTs. (A). Modulation contrast time lapse of the formation of PPLLs after spreading on fibrinogen (t = 00:00:00). PPLLs form in D723H but not in WT cells. Bar 45 μm. (B) Low and high magnification of MT bundles coiling in a D723H derived swelling. Representative images shown were acquired by scanning electron microscopy of extracted D723H cells spread on fibrinogen. Bars 2.5 μm. (C-E) High speed wide field fluorescence imaging. (C) mCherry-Lifeact and GFP-EB3 comets in D723H cell-derived cytoplast were acquired every 5 min. A single slice is shown at different time points as indicated. Bar is 10 μm (D) Images of the behavior of coiled MTs with fluorescent SiR-Tubulin in a D723H dependent swelling is shown at indicated times. Bar is 7 μm. (E) low and high magnification images of GFP-EB3 comets in a D723H cell-derived barbell platelet acquired every second, MIP of 5 planes is shown. Bar is 10 μm. (PDF 3106 kb) [file 12915_2018_584_MOESM1_ESM.pdf]

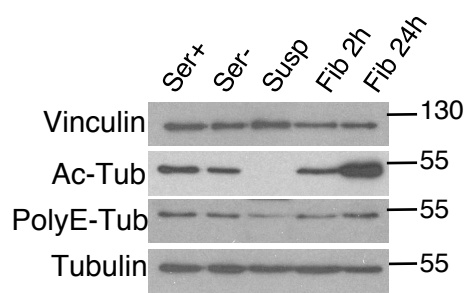

Suppl. Figure 2

Supplement: Supplementary file 9 — Figure S2. Pattern of Ac-and PolyE-MTs in D723H cells. Western blot of D723H CHO cell lysates. Lysates were prepared from adherent cells grown in presence of 10% fetal calf serum (Ser+) or serum starved for two hours (Ser-), from cells in suspension after two hours serum starvation (Susp), from suspended cells spread on fibrinogen for 2 h (Fib 2 h) or 24 h (Fib 24 h). Lysates were successively probed with antibodies against Ac-Tub, PolyE-Tub, total tubulin and vinculin as a loading control. Molecular weight markers in kilodaltons are indicated on the right. (PDF 83 kb) [file 12915_2018_584_MOESM9_ESM.pdf]

A

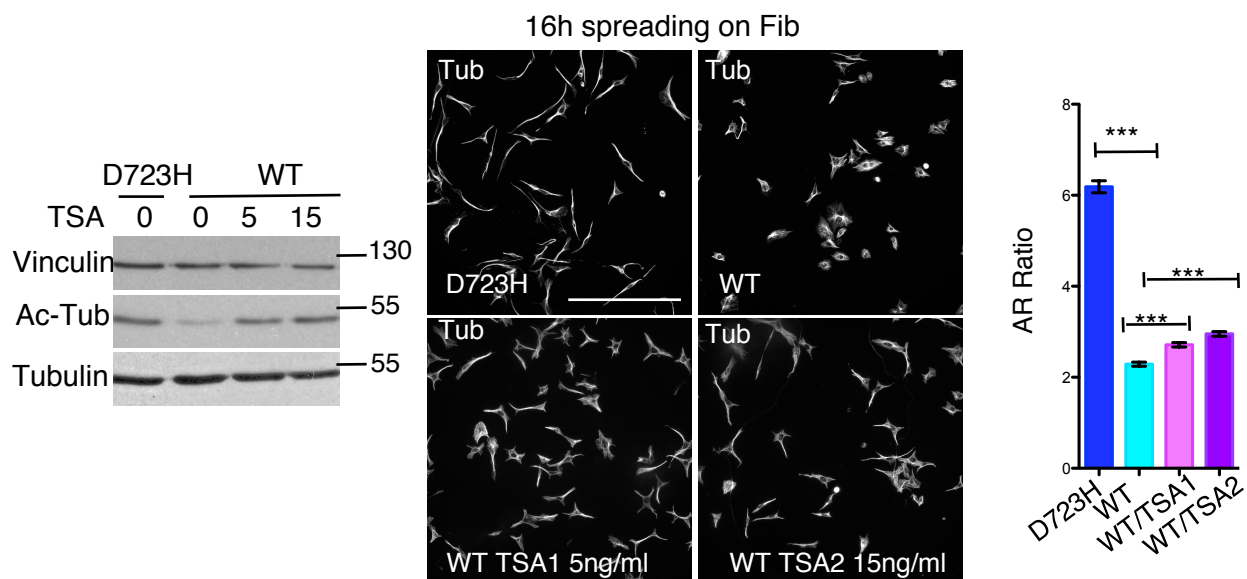

B

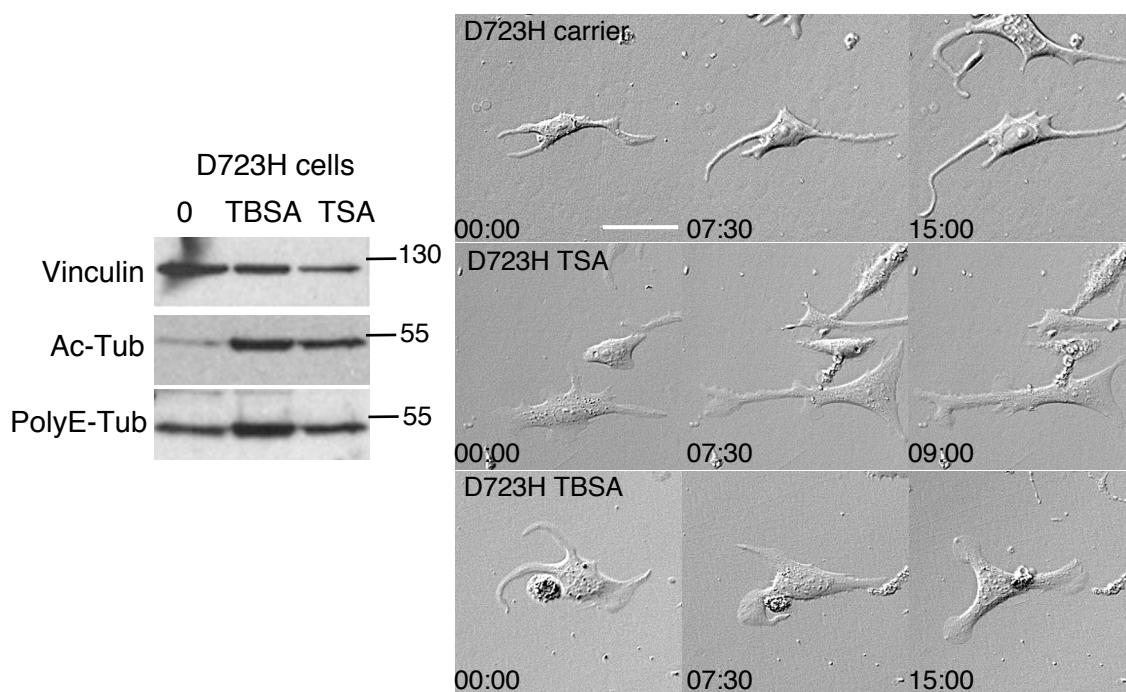

C

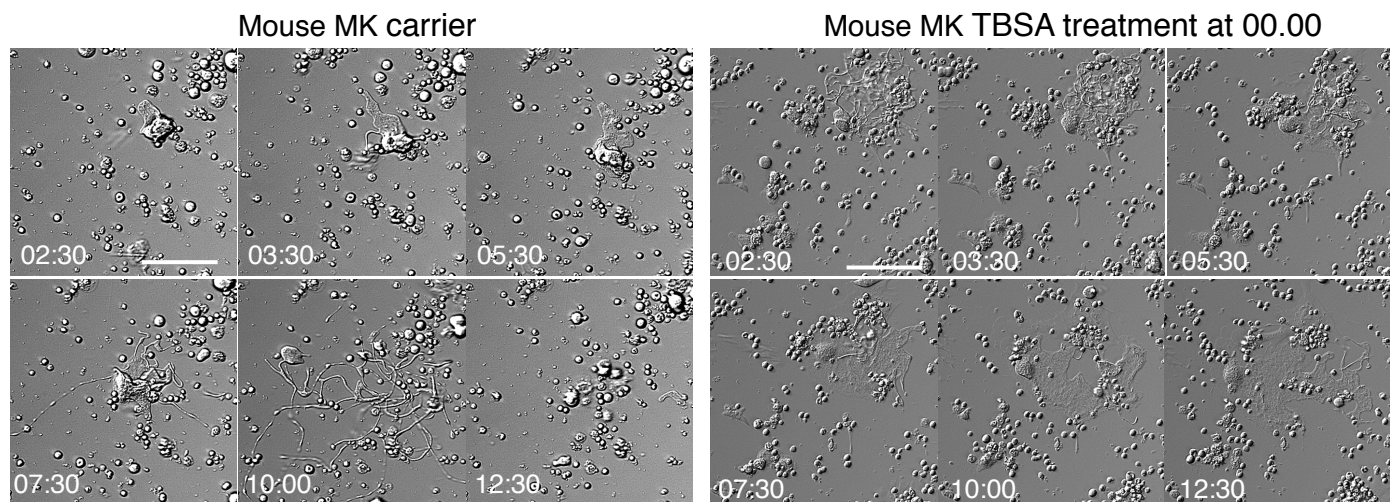

Suppl. Figure 3

Supplement: Supplementary file 10 — Figure S3. Increasing MT acetylation in WT cells is not sufficient to induce PPLL elongations. (A) WT cells treated with low TSA drug concentrations (ng/ml) and spread for 16 h on fibrinogen were analyzed for increased total acetylation level by western blot and for induction of PPLL extensions by immunofluorescence. Representative low magnification and wide field acquired tubulin stained images are shown to visualize cell shape (Bar, 150 μm). Quantification of the AR ratio: n = 3, at least 200 cells per condition were analyzed. Graph shows mean AR ratio, unpaired student t-test, Two tailed ***P < 0.0001. Error bars are SEM (B-C) Modulation contrast time lapse. Cell edges were enhanced using the ImageJ software process enhance contrast function (8%). (B) D723H cells treated with carrier, TBSA (10 μM) or TSA (100 ng/ml) were analyzed for increased Ac-MTs level by Western blot (6 h drug treatment) and for PPLL behavior by time lapse. Bar 50 μm. In drug treated cells, induced PPLL tend to retract and widen with time. (C) Mouse liver-derived-megakaryocytes spread on fibrinogen are treated with carrier or TBSA. Bar 80 μm. As observed on D723H cells, TBSA treatment induced retraction and widening of induced proplatelet (n = 2, 15 megakaryocytes). For better visualization please see corresponding movies. (PDF 9633 kb) [file 12915_2018_584_MOESM10_ESM.pdf]

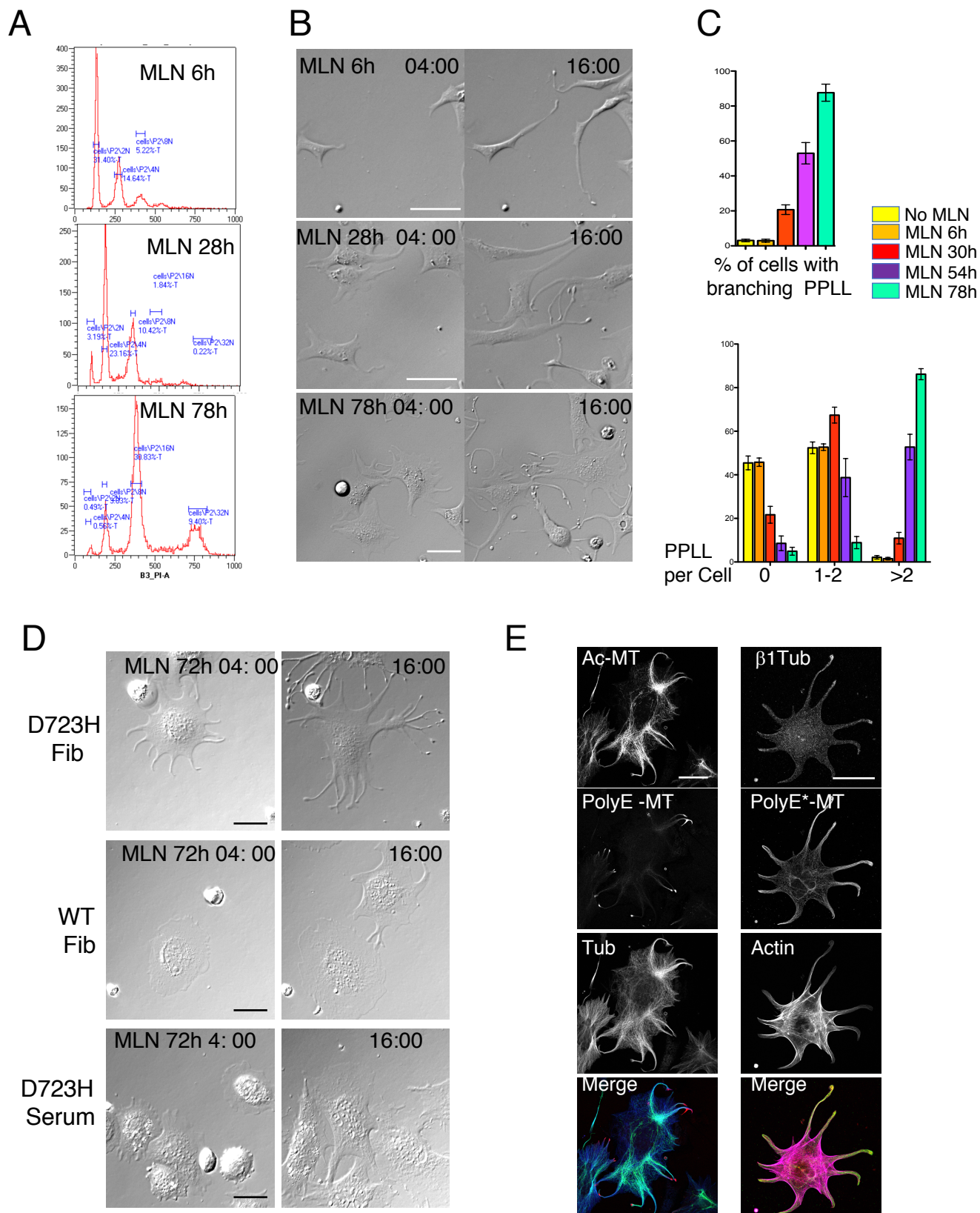

Suppl. Figure 4

Supplement: Supplementary file 13 — Figure S4. D723H polyploidization increases number and branching of PPLLs when spread on fibrinogen. (A-C) D723H cells treated with MLN8237 (400 nM) for indicated times. Cells were analyzed for DNA content by flow cytometry (A) or spread on fibrinogen for 4 h before analyzing their behavior by modulation contrast time-lapse microscopy. (B, bar is 30 μm). (C) Quantification of PPLL numbers and branching in D723H polyploid cells treated as in (A-B) as a function of duration of MLN treatment and spread for 16 h on fibrinogen. MLN8237 treatment increases D723H cells ploidy and their capacity to extend more PPLLs bearing more branchings and more swellings. n = 3 at least 50 cells per condition were analyzed, error bars are SEM. (D) D723H or WT cells treated with MLN8237 (400 nM) for 72 h before spreading on fibrinogen or cultured with serum as indicated, and analyzed by modulation contrast time-lapse microscopy. Nor WT cells spread on fibrinogen nor D723H cells cultured with serum do produce PPLLs, a phenotype observed only in D723H cells spread on fibrinogen. Bar is 50 μm. (E) Representative MIP images of polyploid D723H cells spread on fibrinogen for 16 h and stained with indicated antibodies. Polyploidization does not modify Ac-, polyE- nor β1 MTs localization in PPLLs. Bar 60 μm. (PDF 4828 kb) [file 12915_2018_584_MOESM13_ESM.pdf]

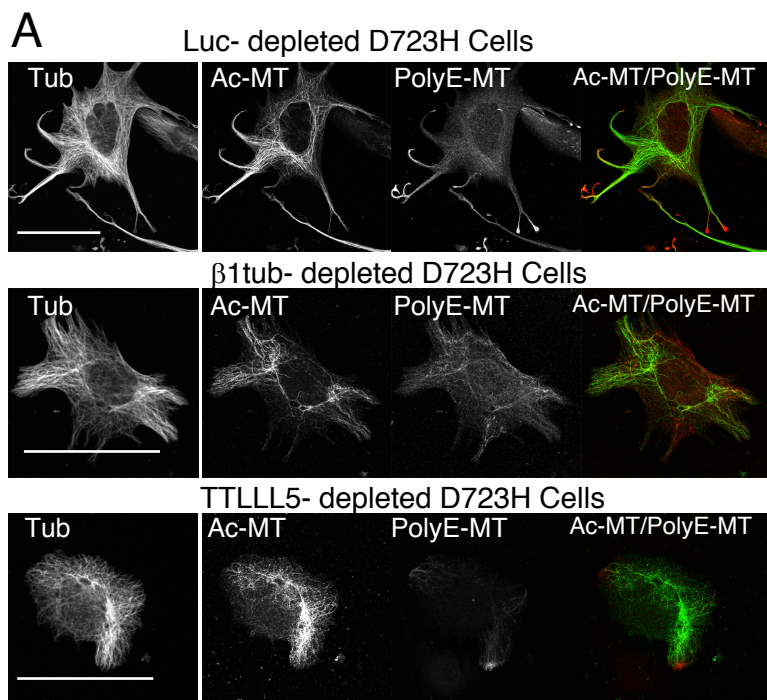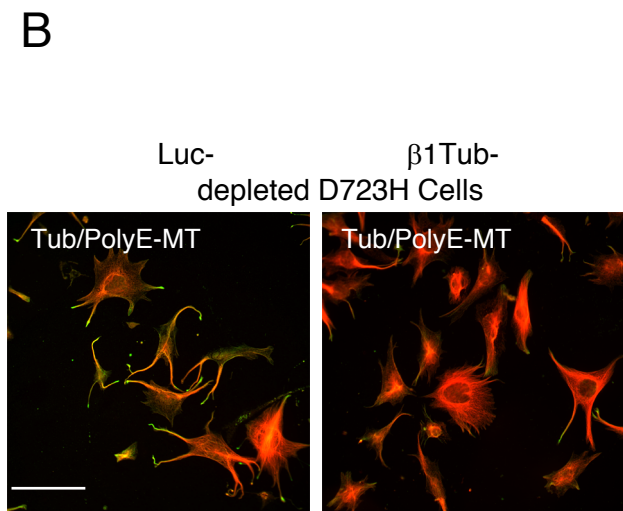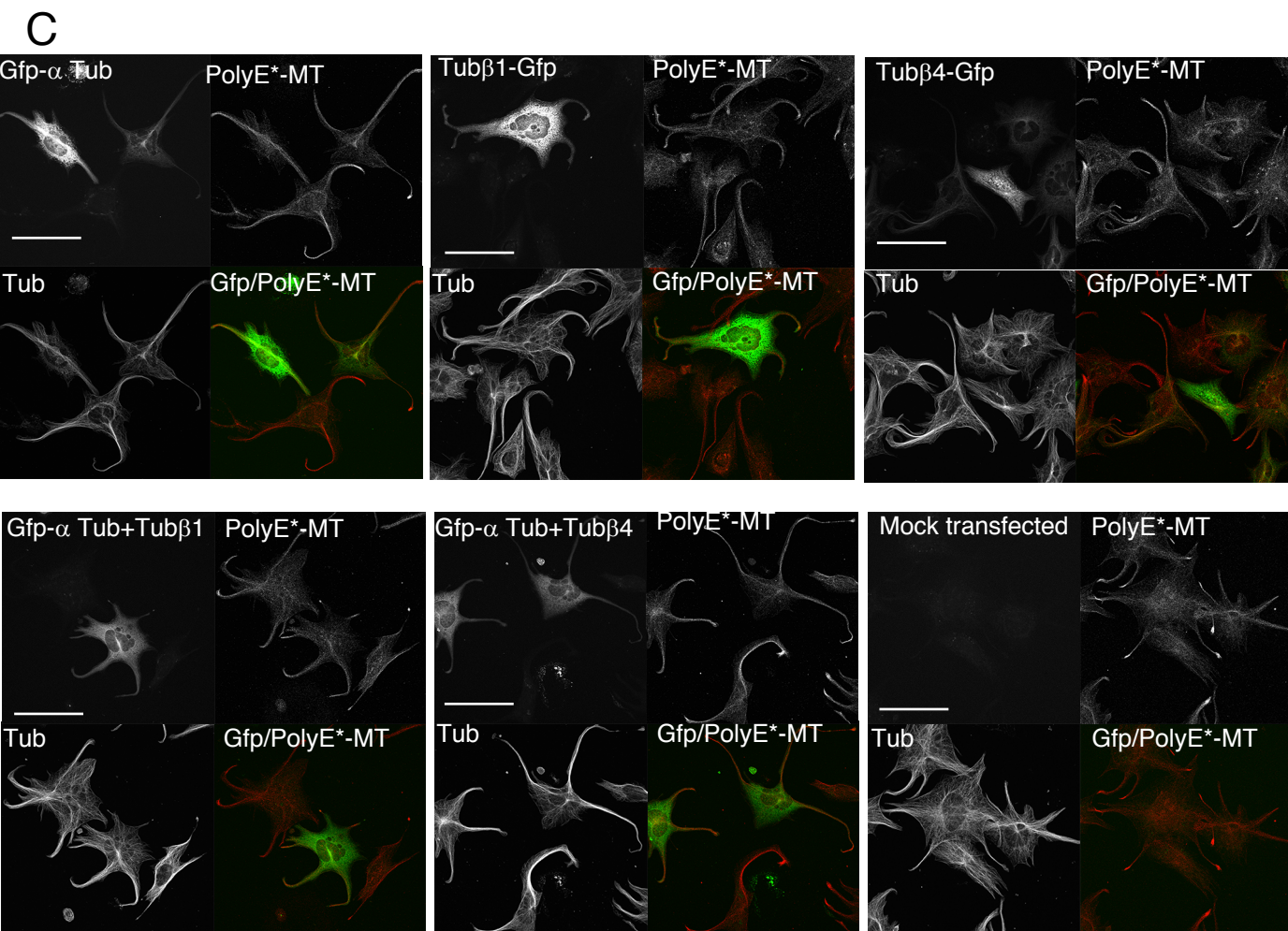

Suppl. Figure 5

Supplement: Supplementary file 15 — Figure S5. Ac-MTs distribution is not affected by loss of β1tubulin or TTLL5. (A) Luc-, β1tubulin- and TTLL5- depleted polyploid D723H cells stained for total MTs, Ac-MTs and PolyE-MTs after 16 h spreading on fibrinogen. Bar is 100 μm. (B) Low magnification of representative merge images of Luc- and β1tubulin- depleted D723H cells stained for total MTs (red) and PolyE-MTs (green) show the cell shape change in β1tubulin depleted cells. Bar is 160 μm. (C) MLN treated and Gfp-α tubulin (αtub) or tubulin β4-Gfp (β4tub) or tubulin β1-Gfp (β1tub) or αtub plus β4tub or αtub plus β1tub transfected D723H cells were analyzed for Gfp expression, total tubulin and PolyE-MTs stainings. Representative images (MIP) are shown. Over 300 overexpressing cells were monitored for each condition. None showed a marked increase in PolyE-MTs staining. Bar 70 μm. (PDF 6370 kb) [file 12915_2018_584_MOESM15_ESM.pdf]
